# Selective single blastocyst transfer reduces the multiple pregnancy rate and increases pregnancy rates: a pre- and postintervention study

**DOI:** 10.1111/j.1471-0528.2007.01584.x

**Published:** 2008-02

**Authors:** Y Khalaf, T El-Toukhy, A Coomarasamy, A Kamal, V Bolton, P Braude

**Affiliations:** a Assisted Conception Unit, Guy’s and St Thomas’ Hospital NHS Foundation Trust London, UK

**Keywords:** Blastocyst transfer, clinical pregnancy, IVF, multiple pregnancy, single embryo transfer pregnancy

## Abstract

**Objective:**

To examine the clinical pregnancy rate (CPR) and multiple pregnancy rate (MPR) in a large *in vitro* fertilisation (IVF) programme before and after the introduction of single blastocyst transfer (SBT) strategy in a selected group of women.

**Design:**

A 3-year pre- and postintervention study.

**Setting:**

A tertiary reproductive medicine and assisted conception unit in a London teaching hospital.

**Population:**

Two thousand four hundred and fifty-one fresh IVF cycles performed between July 2004 and June 2007 at the Assisted Conception Unit at Guy’s and St Thomas’ Hospital NHS Foundation Trust were included in the study.

**Methods:**

In January 2006, we implemented a multidisciplinary intervention involving the introduction of a selective day 5 SBT service together with an educational programme on the risks of multiple pregnancy and potential advantages of blastocyst transfer aimed at couples at high risk of multiple pregnancy.

**Main outcome measures:**

The CPR per cycle started and MPR per clinical pregnancy achieved.

**Results:**

A statistically significant increase in the CPR from 27% (324/1198) to 32% (395/1253) (risk difference [RD] 5%, risk ratio [RR] 1.17, 95% CI 1.03–1.32, *P* = 0.015) and reduction in the MPR per clinical pregnancy from 32% (103/272) to 17% (69/395) (RD 15%, RR 0.46, 95% CI 0.35–0.60, *P* < 0.001) were observed after introduction of the SBT service.

**Conclusion:**

Selective SBT in women with good prognosis can reduce the MPR after IVF while maintaining the overall success rate of the IVF programme.

*Please cite this paper as:*Khalaf Y, El-Toukhy T, Coomarasamy A, Kamal A, Bolton V, Braude P. Selective single blastocyst transfer reduces the multiple pregnancy rate and increases pregnancy rates: a pre- and postintervention study. BJOG 2008;115:385–390.

## Introduction

IVF treatment is well established in the contemporary management of infertility and currently accounts for 1% of the total births in the UK.^1^ Due to the practice of transferring more than one embryo to maximise the chance of pregnancy, the incidence of multiple pregnancy after IVF treatment has risen to over 25%, nearly 20 times higher than would be expected after spontaneous conception.^2^ Compared with singletons, twins have a seven-fold increase in neonatal mortality and six-fold increase in the risk of cerebral palsy.^3^ Maternal morbidity and mortality rates and costs to the health service are also increased.^3^–^6^ As a result, multiple pregnancy is now considered the single most important risk of IVF treatment.^7^–^9^

It has been recognised that the only effective method to reduce the multiple pregnancy rate (MPR) after IVF is to adopt a policy of single embryo transfer (SET).^10^–^13^ An expert panel commissioned by the Human Fertilisation and Embryology Authority in 2005 to review the evidence on multiple births and SET proposed that IVF clinics should develop criteria for selecting groups of women who should be offered SET.^10^ However, guidance on how to implement this policy or its potential effect on a clinic’s overall results was not provided.

A potential risk to implementation of an SET policy is the decline in pregnancy rate per IVF cycle started.^14^,^15^ A Cochrane Review published in 2004^15^ showed that transfer of a single cleavage-stage (day 2 or 3) embryo versus two cleavage-stage embryos resulted in a reduction in the MPR (risk ratio [RR] 0.12, 95% CI 0.03–0.43, *P* = 0.0008), but with a concurrent reduction in the clinical pregnancy rate (CPR) (RR 0.68, 95% CI 0.52–0.9, *P* = 0.006).

Recent evidence suggests that transfer of a single blastocyst on day 5 of *in vitro* culture is associated with a higher CPR compared with transfer of a single cleavage-stage embryo.^16^ Extension of culture until day 5 aids the identification of those embryos likely to have the highest implantation and pregnancy potential.^17^ These two studies,^16^,^17^ however, were restricted to a highly selected group of women with good prognosis, whereas the wider implications of adopting this policy on the overall results in an IVF programme have not been studied.^18^,^19^

In this study, we aimed to examine the impact of implementing a single blastocyst transfer (SBT) strategy in a selected group of women with good prognosis on the effectiveness of a large IVF programme. We hypothesised that the CPR would be maintained and the MPR would be significantly reduced.

## Materials and methods

The study was conducted at the Assisted Conception Unit at Guy’s and St Thomas’ Hospital; a tertiary referral centre performing around 1000 IVF and intracytoplasmic sperm injection (ICSI) cycles per year.

### Study population

Between July 2004 and June 2007, all IVF/ICSI cycles performed in our unit were included. These cycles were divided into two groups—July 2004 to December 2005 (*n* = 1198) and January 2006 to June 2007 (*n* = 1253)—based on the embryo culture and transfer strategy during each period. Cycles involving preimplantation genetic diagnosis or the use of donated oocytes or cryopreserved embryos were excluded. Each couple gave written informed consent to the use of their data for analysis upon entering our IVF programme.

### Ovarian stimulation and IVF/ICSI

Our protocol for ovarian stimulation has been described elsewhere.^20^ Human chorionic gonadotrophin (hCG) 6500 iu (Ovitrelle; Serono Ltd, Middlesex, UK) was administered to induce oocyte maturation when at least three follicles had reached a mean diameter of ≥18 mm. Transvaginal oocyte retrieval was carried out 35–36 hours later using an ultrasound scanner with a 6.5 MHz probe (Hitachi EUB 525; Hitachi, Tokyo, Japan).

For IVF cycles, oocytes were examined for evidence of normal fertilisation 16–18 hours after insemination, with the identification of two pronuclei. For ICSI cycles, following removal of cumulus/corona cells, mature (metaphase II) oocytes were injected with a single, immobilised spermatozoon and examined for survival and normal fertilisation 16 hours after injection. Culture of gametes and embryos was carried out using SAGE culture media (Rochford Medical, Oxford, UK) under oil at 37°C in an atmosphere of 5% CO_2_ in air.

### Embryo culture and transfer strategies

Between July 2004 and December 2005 (2004/2005) up to three cleavage-stage embryos were transferred to the uterus 2–3 days after insemination using an Edwards–Wallace embryo transfer catheter (Sims Portex Ltd, Hythe, Kent, UK). Between January 2006 and June 2007 (2006/2007), cycles in which there were a minimum of four 8-cell cleavage-stage embryos with less than 10% cytoplasmic fragmentation^21^,^22^ on day 3 were offered extended embryo culture until day 5 to allow further development of these embryos and transfer of a single blastocyst if a high-quality blastocyst^17^ was present. We continued to offer transfer of up to three embryos for women where the cohort of embryos did not satisfy these criteria. Apart from the introduction of extended embryo culture and SBT for the women with good prognosis, no other changes in our clinical protocols took place.

All women who underwent embryo transfer received supplemental progesterone pessaries (Cyclogest; Shire Pharmaceuticals Ltd, Hants, UK) 400 mg daily throughout the luteal phase and until 8 weeks of gestation if pregnancy occurred.

### Patient education

To allow for the successful implementation of the SBT strategy, audiovisual and written educational information regarding the risks of multiple pregnancy and advantages of SBT was given to all women at a monthly patient information seminar as well as during one to one consultations. The rationale behind our strategy was also displayed on a wall poster located in the participants’ waiting area, together with regularly updated results. Women who were eligible for extended embryo culture and SBT were contacted on day 3 of culture to ensure they understood their options and to discuss any remaining concerns they might have.

### Outcome measures

The study outcome measures were the CPR per cycle started and MPR per clinical pregnancy achieved. Pregnancy was confirmed by a positive urine hCG test 16 days after oocyte retrieval. A clinical pregnancy was defined as the presence of fetal heart activity detected by ultrasound 3 weeks after the positive pregnancy test. Implantation rate was defined as the number of gestational sacs observed on ultrasound scanning divided by the number of embryos transferred.

### Statistical analysis

In this centre, IVF/ICSI cycle data are prospectively collected and stored in a relational database (FileMaker Pro 6.4; FileMaker Inc, Santa Clara, CA, USA). Cycles performed in 2004/2005 (before implementation of the SBT strategy) were compared with those performed in 2006/2007 (when SBT strategy was introduced). Statistical analysis was performed using *t* test or Mann–Whitney *U* test for continuous variables as appropriate and chi-square test for discrete variables. Statview software package (Abacus Concepts Ltd, Berkeley, CA, USA) was used for statistical analysis. Statistical significance was set at *P* < 0.05.

## Results

During the entire study period, 2451 fresh IVF/ICSI cycles were started, 2086 (85%) cycles reached embryo transfer and 3890 embryos were replaced (mean of 1.86 ± 0.46 embryo per transfer). The overall CPR per cycle started was 29.3% (719/2451) and the MPR per clinical pregnancy was 24% (172/719) (Table 1). The CPR was similar in cycles in which IVF (*n* = 1076) or ICSI (*n* = 1375) was used for oocyte insemination (30 versus 29%, respectively, RR 1.05, 95% CI 0.93–1.19, *P* = 0.5).

**Table 1:** Characteristics of treatment cycles performed during the study period

Characteristic	2004/2005 (*n* = 1198)	2006/2007 (*n* = 1253)	Difference (95% CI)	*P* value
Age (years)	35.2 (4.5)	35.8 (4.4)	−0.59 (−0.94 to −.0.24)	0.001
IVF cycle order	1.7 (1.0)	1.7 (1.1)	0.016 (−0.1 to 0.069)	0.71
Basal FSH level (iu/l)	6.5 (2.4)	7.3 (2.7)	−0.82 (−1.03 to −0.62)	0.0001
Basal estradiol level (pmol/l)	173 (159)	169 (110)	4.1 (−7.4 to 15.6)	0.49
Duration of stimulation, days	10.1 (1.7)	10.4 (1.9)	−0.29 (−0.43 to −0.15)	0.0001
Daily dose of FSH (iu)	281 (105)	281 (101)	0.24 (−7.9 to 8.4)	0.95
Number of oocytes collected	10.3 (6.6)	11.2 (7.4)	−0.84 (−1.4 to −0.27)	0.004
Cycles reaching ET (%)	83	87		0.004
Day 5 selective SBT (%)	––	9		<0.0001
Number of embryos replaced per transfer	1.9 (0.4)	1.8 (0.5)	0.07 (0.03 to 0.11)	0.0002
Cycles achieving freezing (%)	22	28		<0.001
Pregnancy rate/cycle (%)	33	38		0.013
CPR/cycle (%)	27	32		0.015
Implantation rate (%)	25	28		0.11
MPR (%)	32	17		<0.001

ET, embryo transfer; FSH, follicle-stimulating hormone; SBT, single blastocyst transfer.

Values are expressed as mean (SD) unless indicated otherwise.

In 2004/2005 (preintervention period), 1198 fresh IVF/ICSI cycles were performed, of which 994 (83%) reached embryo transfer. The CPR per cycle started was 27% (324/1198) and the MPR was 32% (103/324) per clinical pregnancy (Table 1). In 2006/2007 (postintervention period), 1253 fresh IVF/ICSI cycles were performed, of which 1092 (87%) reached embryo transfer. The CPR per cycle started was 32% (395/1253) and the MPR was 17% (69/395) per clinical pregnancy.

### Day 5 culture and blastocyst transfer

All cycles that had extended embryo culture had at least one blastocyst available for transfer on day 5. The percentage of IVF/ICSI cycles having a blastocyst transfer in the study increased from 0% (0/1198) in 2004/2005 to 17% (211/1253) per cycle started and 19% (211/1092) per embryo transfer in 2006/2007. The percentage of cycles opting to have a blastocyst transfer also increased from 16% of transfers (82/521) between January and September 2006 to 23% (129/571) between October 2006 and June 2007 (RR 1.5, 95% CI 1.1–1.9, *P* = 0.003). Of the 211 blastocyst transfers performed in the study, 137 (65%) were SBT.

### Single embryo transfer

The percentage of cycles having SET in the study increased from 13% per embryo transfer (127/994) in 2004/2005 to 23% (250/1092) per transfer in 2006/2007 (RR 1.8, 95% CI 1.5–2.2, *P* < 0.001). The proportion of IVF cycles where a single embryo was transferred electively and surplus embryos cryopreserved altered significantly from 1.9% of all cycles achieving surplus embryos cryopreservation (5/263) in 2004/2005 to 38% (129/342) in 2006/2007 (RR 21.9, 95% CI 9.1–52.7, *P* <0.001). Of the 129 elective SET in 2006/2007, 118 (92%) were elective SBT on day 5. Consequently, the mean number of embryos replaced per transfer decreased in the entire IVF programme from 1.9 ± 0.4 embryos in 2004/2005 to 1.8 ± 0.5 embryos (*P* < 0.001). As a result of transferring fewer embryos, the proportion of cycles achieving cryopreservation of surplus embryos increased from 22% (267/1198) to 28% (341/1224, RD = 6%, RR 1.3, 95% CI 1.09–1.44, *P* = 0.0015).

### Analysis of outcome measures

#### Clinical pregnancy rate

The overall CPR per cycle started was higher in 2006/2007 (32%, 395/1253) than in 2004/2005 (27%, 324/1198, RD = 5%, RR 1.17, 95% CI 1.03–1.32, *P* = 0.015). The improvement in the CPR was observed in cycles where women’s age was less than 40 years (35% [352/1014] versus 30% [303/1026], RD = 5%, RR 1.18, 95% CI 1.04–1.33, *P* = 0.012) and in those where women’s age was 40 years or more (18% [43/239] versus 12% [21/172], RD = 6%, RR 1.5, 95% CI 0.91–2.39, *P* = 0.11, Table 2). The increase in the CPR was due to a higher CPR per transfer (49%, 104/211) in the group of women who had a blastocyst transfer. The CPR in cycles where day 2 or 3 cleavage-stage embryos were transferred was similar in 2004/2005 and 2006/2007 (32.6 versus 33%, respectively, RR 1.01, 95% CI 0.89–1.15, *P* = 0.84).

**Table 2:** CPR and MPR in different age groups before and after introducing SBT service

Age group (years)	CPR (%)		*P* value	MPR (%)		*P*value
				
	Before SBT (2004/2005)	After SBT (2006/2007)		Before SBT (2004/2005)	After SBT (2006/2007)	
<35	35 (185/527)	41 (202/496)	0.06	38 (71/185)	19 (38/202)	<0.0001
35–37	27 (88/332)	33 (101/309)	0.08	25 (22/88)	19 (19/101)	0.30
38–39	18 (30/167)	23 (49/209)	0.19	23 (7/30)	14 (7/49)	0.30
≥40	12 (21/172)	18 (43/239)	0.11	14 (3/21)	12 (5/43)	0.76
**Total**	27 (324/1198)	32 (395/1253)	0.015	32 (103/324)	17 (69/395)	<0.001

Values are expressed as percentages.

#### Multiple pregnancy rate

**Figure 1:**
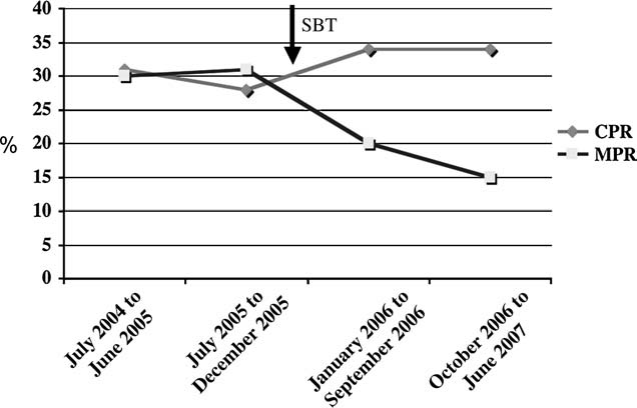
Effect of introduction of SBT policy.

The MPR throughout the study period decreased by 47% from 32% (103/324) in 2004/2005 to 17% after introduction of the SBT strategy in 2006/2007 (69/395, RD = 15%, RR 0.46, 95% CI 0.35–0.60, *P* <0.001) (Table 2 and Figure 1). Most of the multiple pregnancies in 2004/2005 (100/103) occurred in cycles performed for women younger than 40 years of age and the risk was highest (41%, 49/121) in cycles where surplus embryos were available for cryopreservation. In 2004/2005, women in this group (women younger than 40 years and surplus embryos available for cryopreservation) were not routinely offered the option of an SET, and in only 2% (4/246) of their transfers, a single embryo was replaced electively. The percentage of elective SET changed significantly in 2006/2007. Of the 316 transfers in the corresponding group in 2006/2007, 40% (125/316) were elective SET. As a result, the MPR in this group dropped by 61% from 41 to 16% (28/174, RD = 25%, RR 0.40, 95% CI 0.27–0.59, *P* < 0.001).

In addition, with the increase in the proportion of cycles that opted for blastocyst transfer from 16% in the first half of 2006/2007 (January to September 2006) to 23% in the second half of 2006/2007 (October 2006 to June 2007, *P* = 0.003), the MPR showed a concomitant (but nonsignificant) reduction from 20% (39/190) to 15% (30/205), respectively (RD 5%, RR 0.72, 95% CI 0.46–1.1, *P* = 0.13, Figure 1).

## Discussion

The goal of any fertility treatment should be the birth of a healthy singleton infant.^10^ Although the effectiveness of SET in reducing the MPR after IVF has been demonstrated,^11^–^13^ the main challenge is to achieve this reduction while maintaining the overall success rate within the entire IVF programme.

Women with good prognosis, defined in our study as those who have at least four 8-cell embryos with less than 10% cytoplasmic fragmentation, not only have the best chance of producing blastocysts on day 5 of culture but are also at greatest risk of multiple pregnancy if two embryos are transferred.^13^,^23^ Extension of embryo culture till day 5 aids the identification and selection of those embryos most likely to implant. As a result, a high pregnancy rate might be expected after transferring a single blastocyst.^16^

To our knowledge, this is the first study to examine the effects of applying an SBT strategy in a selected group of good prognosis women on the overall results of a large IVF programme. We compared the CPR per cycle started and the MPR per clinical pregnancy achieved during an 18-month period before and after introduction of the SBT service in our IVF programme. The objective from introducing this service was to significantly reduce the MPR by at least one-third,^5^ thereby reducing the morbidity and mortality of babies and mothers.

The present study provides evidence that education and appropriate selection of participants can achieve a reduction in the MPR by almost 50% without compromising the effectiveness of the IVF programme. In fact, we observed an increase in the CPR after introduction of the SBT service despite an increase in women’s mean age and basal follicle-stimulating hormone level, mainly due to identification of embryos with the highest implantation potential through extended culture.^16^,^17^ The increase in the CPR attests to the significant potential of elective SBT to reduce the risk of multiple pregnancy when applied judiciously. Although only one in 11 (118/1253) cycles started in 2006/2007 had an elective SBT, a dramatic reduction in the MPR was achieved since only cycles at high risk of multiple pregnancy were selected.

Our data should, therefore, encourage other IVF programmes to develop similar strategies to lower their MPR without fear of reduction in their IVF success rates. This reassurance is particularly relevant in countries where the majority of IVF treatment cycles are not state funded, mounting more pressure on the treating physician to maximise each patient’s chance of pregnancy per cycle.^24^–^27^ Only 19% of cycles reaching embryo transfer satisfied our eligibility criteria for extended culture and blastocyst transfer. In the next phase of our strategy to further reduce the MPR, we intend to offer extended culture and SBT in more cycles, by including those with three (rather than four) 8-cell cleavage-stage embryos with less than 10% cytoplasmic fragmentation on day 3 of culture. Other clinics need to examine their patient’s characteristics and laboratory performance and modify their selection criteria accordingly.^24^ Our experience could also pave the way for the development of agreed national guidelines for SET, similar to those already in place in several European countries.^28^

Another advantage to this strategy is the increase in the proportion of cycles where supernumerary embryos are available for cryopreservation. By replacing fewer embryos, and at a stage where quality is deemed to be better, it is likely that a larger proportion of cycles would have surplus embryos suitable for cryopreservation. In our study, the percentage of cycles achieving embryo cryopreservation increased by 30% (from 22 to 28%) after introducing the SBT service, thereby maximising the cumulative chance of pregnancy from a single cycle of ovarian stimulation and oocyte retrieval.^12^ The presence of a successful blastocyst freezing–thawing programme would further encourage women to choose the option of SBT and cryopreserve supernumerary embryos.^26^,^29^

Finally, patient acceptability is central to the success of any treatment modality.^30^ There is evidence that multiple pregnancy is a frequently desired outcome of treatment by infertile women.^31^,^32^ It was therefore critical in our strategy to ensure that women are fully informed about the benefits of SBT and risks of twin pregnancy and to promote a safe practice of SET. Audiovisual and written information was disseminated to our women for this purpose, leading to sustained increase in patient acceptability to the SBT policy in 2006/2007. This paradigm shift in acceptance of SET concurs with recent research highlighting the importance of couples’ education in changing their attitude towards what is regarded as a desired IVF treatment outcome.^33^

In conclusion, introducing an SBT service aimed at women with good prognosis has enabled us to almost half the MPR and improved the overall efficiency of the entire IVF programme. The key elements behind the success of this strategy were selection and education of women at high risk of multiple pregnancy.
